# Effect of Personalized Email-Based Reminders on Participants’ Timeliness in an Online Education Program: Randomized Controlled Trial

**DOI:** 10.2196/43977

**Published:** 2023-10-13

**Authors:** Olle Bälter, Andreas Jemstedt, Feben Javan Abraham, Christine Persson Osowski, Reuben Mugisha, Katarina Bälter

**Affiliations:** 1 Division of Media Technology and Interaction Design Kungliga Tekniska Högskolan - Royal Institute of Technology Stockholm Sweden; 2 Department of Public Health Sciences Mälardalen University Västerås Sweden; 3 Department of Medical Epidemiology and Biostatistics Karolinska Institutet Stockholm Sweden

**Keywords:** online learning, personal reminders, timeliness, self-regulated learning, adult education, education, students, learning, email, online, tool, intervention, program

## Abstract

**Background:**

Postsecondary students need to be able to handle self-regulated learning and manage schedules set by instructors. This is particularly the case with online courses, as they often come with a limited number of social reminders and less information directly from the teacher compared to courses with physical presence. This may increase procrastination and reduce timeliness of the students. Reminders may be a tool to improve the timeliness of students’ study behavior, but previous research shows that the effect of reminders differs between types of reminders, whether the reminder is personalized or general, and depending on the background of the students. In the worst cases, reminders can even increase procrastination.

**Objective:**

The aim of this study was to test if personalized email reminders, as compared to general email reminders, affect the time to completion of scheduled online coursework. The personalized reminders included information on which page in the online material the participants ought to be on at the present point in time and the last page they were on during their last session. The general reminders only contained the first part of this information: where they ought to be at the present point in time.

**Methods:**

Weekly email reminders were sent to all participants enrolled in an online program, which included 39 professional learners from three East African countries. All participants in the Online Education for Leaders in Nutrition and Sustainability program, which uses a question-based learning methodology, were randomly assigned to either personalized or general reminders. The structure of the study was AB-BA, so that group A received personalized reminders for the first unit, then general reminders for the rest of the course, while group B started with general reminders and received personalized reminders only in the third (and last) unit in the course.

**Results:**

In total, 585 email reminders were distributed, of which 390 were general reminders and 195 were personalized. A Bayesian mixed-effects logistic regression was used to estimate the difference in the probability of being on time with one’s studies. The probability of being on time was 14 percentage points (95% credible interval 3%-25%) higher following personalized reminders compared to that following general reminders. For a course with 100 participants, this means 14 more students would be on time.

**Conclusions:**

Personalized reminders had a greater positive effect than general reminders for a group of adults working full-time while enrolled in our online educational program. Considering how small the intervention was—adding a few words with the page number the student ought to be on to a reminder—we consider this effect fairly substantial. This intervention could be repeated manually by anyone and in large courses with some basic programming.

## Introduction

Although online learning has great potential, it is also associated with challenges of student self-regulated learning that are associated with self-control [1] and the ability to plan, prioritize, and manage schedules set by online instructors [2]. For online learners to thrive, instructors need to identify nudges that support learners who struggle with self-regulated learning [2]. Online instructors who apply online behavior management techniques, such as providing reminders on assignment deadlines, may achieve improved online learning outcomes [3].

Other challenges with online learning include communication by teachers about assignments and deadlines. Whereas teachers in traditional classroom settings have the option to inform students about assignments orally, this information is often given solely in writing in online courses [4]. Written reminders may therefore serve as a nudge for students to complete their tasks in an online course. A review of various nudging approaches in education, including reminders, showed that reminding students to complete a certain task often has positive effects, especially among students with low socioeconomic status [5]. However, it is not clear if the effect of reminders is short-lived or if the effect of repeated reminders may decrease over time. Nudging in general may also impede intrinsic motivation or leave individuals feeling pressured to perform a behavior [5]. Thus, to avoid negative effects, it is important to tread carefully when implementing routines for reminders.

According to social comparison theory, people compare themselves to others to establish their personal worth [6]. This was tested on massive open online course (MOOC) students to increase their completion rates by sending them information that compares their performance with previously successful learners, and it worked well, but only for already highly educated learners [7]. In another social comparison study, MOOC students were divided into three groups: the first group received a positively formulated email (you did better than n% of students), the second group received a negatively formulated email (you did worse than n% of students), and the third group served as a control and did not receive any emails. Both test groups improved their test scores by 8% compared to the control group. The positive emails had the biggest effect on students who already did well in the course, and the negative emails on those who performed poorly [8]. Moreover, in a blended university course, email reminders increased the number of completed quizzes. The effect was strongest for the simplest form of reminder (ie, an email with text information regarding the deadline for the next quiz). More advanced and personalized email reminders in the form of graphs on individual performances and study advice were more effective than the control group, but not as effective as the simple reminders [9].

How successful reminders are may depend on how they are designed and adjusted to the cultural context of the students. The aim of this study was to test if personalized email reminders, as compared to general email reminders, in a program targeting adult participants affected the timeliness of the completion of their online coursework. The Online Education for Leaders in Nutrition and Sustainability (OneLearns) program in which this study is embedded is an online program targeting government officials and decision makers in nongovernmental organizations (NGOs) in Rwanda, Ethiopia, and Kenya and covers the sustainable development goals with a focus on effective digital learning, digital health literacy, and nutrition.

## Methods

### The OneLearns Program

The online OneLearns program uses a question-based learning methodology developed within the Open Learning Initiative at Carnegie Mellon University, United States. The program was organized by teachers and researchers at the KTH Royal Institute of Technology and Mälardalen University, both in Sweden, covering the domains of digital learning, digital health literacy, sustainable development, public health, and nutrition. To ensure that the program would be relevant for the target participants from Rwanda, Ethiopia, and Kenya, the content was discussed with research colleagues from East Africa. The OneLearns program was developed in line with best practices for online learning [10]. The first version of OneLearns from 2020 to 2021 has been described in detail in *JMIR Formative Research* [11], and here we present a reminder study performed during the second installment of the program.

### Recruitment Process

The online recruitment process ran between June and August 2021, targeting potential participants from various ministries with a focus on the ministries of health, education, and agriculture, and NGOs with public health, nutrition, and/or education in their missions. We collaborated with the embassies of Rwanda, Kenya, and Ethiopia in Stockholm, who distributed the call for applications in their respective countries. To reach NGOs, we used personal networks and correspondence with the Swedish embassies in the target countries.

We received 78 applications, including 40 from Ethiopia, 31 from Rwanda, and 7 from Kenya. Applicants were screened according to educational background, current job position, and English skills. Furthermore, we aimed to achieve a gender balance as close to a 1:1 ratio of male to female participants as possible and a pool of participants from various backgrounds in the pertinent sectors. However, the number of female applicants was only 15. For this reason, for admission to the last few positions, rather than randomly selecting among candidates with the same score, female applicants were prioritized by being given an extra “point” during the scoring process after the top candidates had been selected based on merit only, regardless of gender. This increased the number of admitted women from 10 to 13. This also ensured that no men with higher merit would be excluded.

Subsequently, there were 47 applicants selected for the second phase of recruitment during which online commitment forms describing in detail the requirements of the course and the expectations from the program were distributed. The form included information about the time commitment, workload, and connectivity requirements, and applicants who agreed to the conditions were expected to sign the agreement form. Out of the 47, there was a final set of 43 participants who signed the agreement form and were enrolled in the program.

Of the 43 participants, 22 were from Ethiopia, 14 were from Rwanda, and 7 were from Kenya. All 43 participants successfully registered online to the program, but 4 were inactive and did not attend any online activities. These 4 were subsequently suspended from the program, leaving 39 active participants. Two of the inactive participants reported having a clash with their work schedule, while the other two did not communicate any reason despite repeated communications.

### Course Delivery and Timeline

**Figure 1:**
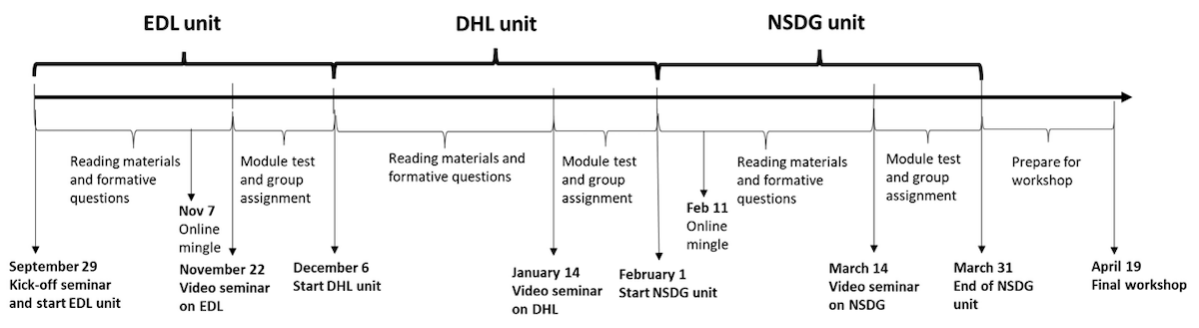
Timeline for the OneLearns program. The course ran between September 29, 2021, and April 19, 2022, and comprised 3 modules: EDL, DHL, and NSDG. DHL: digital health literacy; EDL: effective digital learning; NSDG: nutrition and the sustainable development goals.

The OneLearns course ran between September 29, 2021, and April 19, 2022 (Figure 1). The course was conducted primarily online, but the final workshop was conducted in a hybrid online and physical format in Kigali, Rwanda. Each participant was expected to spend approximately 40 hours of study time during the program, and the program design allowed for a self-paced learning approach to a large extent.

The OneLearns program consisted of 3 units and a final workshop. Each unit was planned to be completed in approximately 2 months. The participants began by working with the interactive online material and answering formative questions and concluded the program with an online video seminar with instructors from Sweden, a group assignment, and an individual online module test with both auto-graded and manually graded questions (for open answers). These group assignments required that all participants be synchronized in their progress through the course, as late stragglers would impede the quality of the group assignment for those who were on time. The program ended with a full-day hybrid workshop in Kigali, which was another reason for encouraging the participants to be synchronized in the course material at that point in time.

In the previous round of the program in 2020-2021 [11], weekly reminders with deadlines, including a general group progress report, were sent to the participants. For the 2021-2022 round, this was replaced by the current reminder study.

### Study Design

The participants were randomly divided into two groups using the randomize function in Microsoft Excel (Microsoft Corporation). All participants received reminders in the form of an email sent out manually by one of the program coordinators at the end of every week. The structure of the study was AB-BA: group A received personalized reminders during the first unit, then general reminders for the rest of the course, while group B started with general reminders and received personalized reminders only for the third and last unit in the course.

Because each unit consisted of topics mostly unrelated to the previous unit and they were equivocally distributed, each unit was treated as a testing period for the reminders. The intervention period only measured the students’ pace of work up to the interactive online seminar. Data on the students’ pace was automatically generated by the course platform, and a program was written to compare the students’ progress with the current date each week (see Multimedia Appendix 1).

Personalized email reminders included information on how many pages the participant needed to cover per week to stay on track, what page the participant should have been on, and what page he or she was currently on. For example, a student on pace to complete the course on time may receive the following email:

Dear “Participant Name”,

We see that you have started working with your course material. To be on track you should be covering at least one to one and half pages per week and today you should be at least on page 20 of the course. You are currently on page 21 which means that you are on track to finish the course material on time.

For a student who is falling behind, the content of the email may be adjusted as follows:

It is good to see that you have started with the course material in the 3rd Unit. However, be aware that in order to have an even workload and finish the Nutrition and Sustainability unit on time we expect you to cover between half to one page per day and you should now be on page 46 of the course. But according to our data you are currently behind on page 42 and not on track to complete the course on time.

The general email reminders only included information about what pace the student should be working at and what page we would expect them to be on to stay on track. The following is an example of a general reminder email:

Dear OneLearns Participant,

To have an even pace and be on track to completing the course material on time, you should be covering at least one to one and half pages per day and today you should be at least on page 20.

### Ethical Considerations

Conducting the OneLearns program and evaluating its results did not require ethical approval according to paragraph 2 of the Swedish Ethical Review Authority. Ethical approval is only necessary if a study collects sensitive personal data from the participants, such as physical procedures, uses a method that will impact the participants physically or physiologically or presents a risk of harm, or involves biological material, which was not the case in this study [12]. Enrollment in this web-based program was voluntary. Participating in the program was regarded as informed consent, and students were free to discontinue the program at any point without stating why.

Information was sent to all participants about the plan to do a study on the use of reminders, though we did not go into the details on how. We informed the participants that they were not obliged to participate in the study and that they may opt out at any time without consequence to their participation in the program.

The data for evaluating the study were deidentified and presented on a group level; as such, it is not possible to identify or trace any individuals. Participation in the program and the study was not compensated, but all participants who finished the program received a certificate.

When planning the reminder study, a potential ethical problem may have arisen if one group received reminders and the other did not, as this could have influenced the likelihood of the control group participants completing the program and receiving the certificate. Therefore, we included the general reminders, though we are cognizant that their inclusion may have masked potential group differences that could have been measured on receiving personalized reminders versus none at all.

### Data Analysis

To investigate how personalized reminders influenced timeliness, the data were analyzed in two ways. First, we computed the absolute difference in probability of being on time in the two reminder conditions. Furthermore, because each participant was reminded multiple times and all participants were reminded at approximately the same time point, we included these additional factors in the analysis. Specifically, we used a Bayesian mixed-effect logistic regression, using a weakly informative prior, with being on time as a fixed effect and participant and reminder time point as random effects; that is, a random intercept was fitted for each participant and reminder time. In other words, besides including the effect of the reminders, the analysis considered individual differences in the probability to be on time and whether it was easier or more difficult to be on time at any of the 15 reminder time points. The effects of the reminders were estimated by calculating the median posterior probability distributions produced by the Bayesian analysis. These point estimates were combined with the corresponding credible intervals (CIs) and visualizations to infer the effects’ sizes and the uncertainty of the estimates. The analyses were conducted using R version 4.1.1 (R Foundation for Statistical Computing) [13] and the Stan computational framework [14]. The regression analysis was done by accessing Stan with the brms package in R [15].

## Results

### Participants

Of the 39 active participants enrolled in the program, a majority were younger than 40 years (n=31, 79%), and the gender distribution was skewed in that only 33% (n=13) were women because there were fewer female applicants. Almost half (n=19, 49%) of the participants were from Ethiopia, 33% (n=13) were from Rwanda, and 18% (n=7) were from Kenya. Approximately a quarter (n=10, 24%) of the participants were working in NGOs, while the rest worked in governmental organizations. Most of the participants had an educational background related to health, such as public health (n=12, 31%), nutrition (n=7, 18%), environmental health (n=2, 5%), and the medical field (n=7, 18%). The remaining participants had a background in agriculture (n=3, 8%) or other fields (n=11, 28%), including sociology, community development, natural resource management, and chemistry. The participants were divided into 2 groups, and there were no significant differences between the 2 groups regarding gender, educational level, nationality, NGO versus government affiliation, or age.

### Reminder Data

In total, 585 email-based reminders were sent out to 39 participants over a period of 5 months. Of these, 390 were general reminders and 195 were personalized.

**Figure 2:**
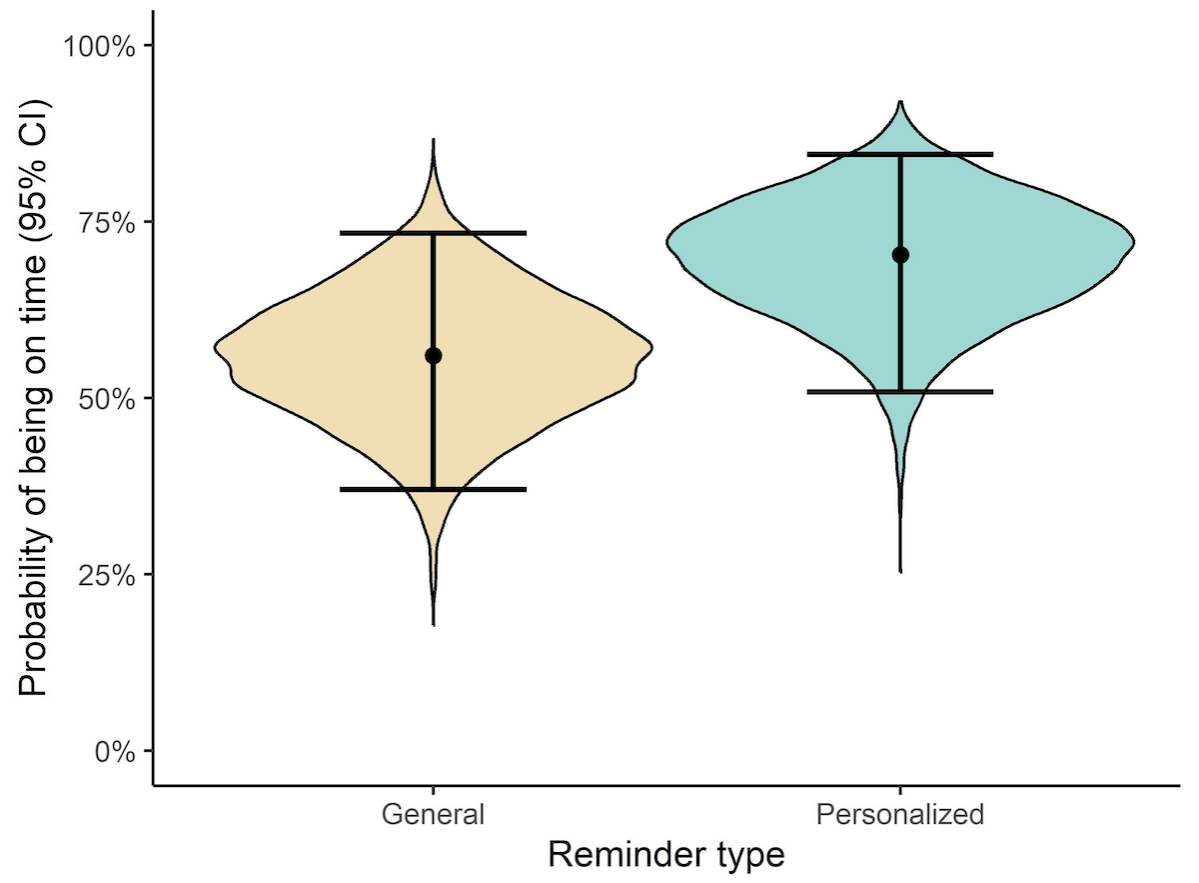
Violin plots, medians, and 95% credible intervals (CI) illustrating the estimated probability to be on time with the course after general and personalized reminders. The width of the violin plots illustrates the relative probability of possible probabilities for being on time following the two reminder types.

**Figure 3:**
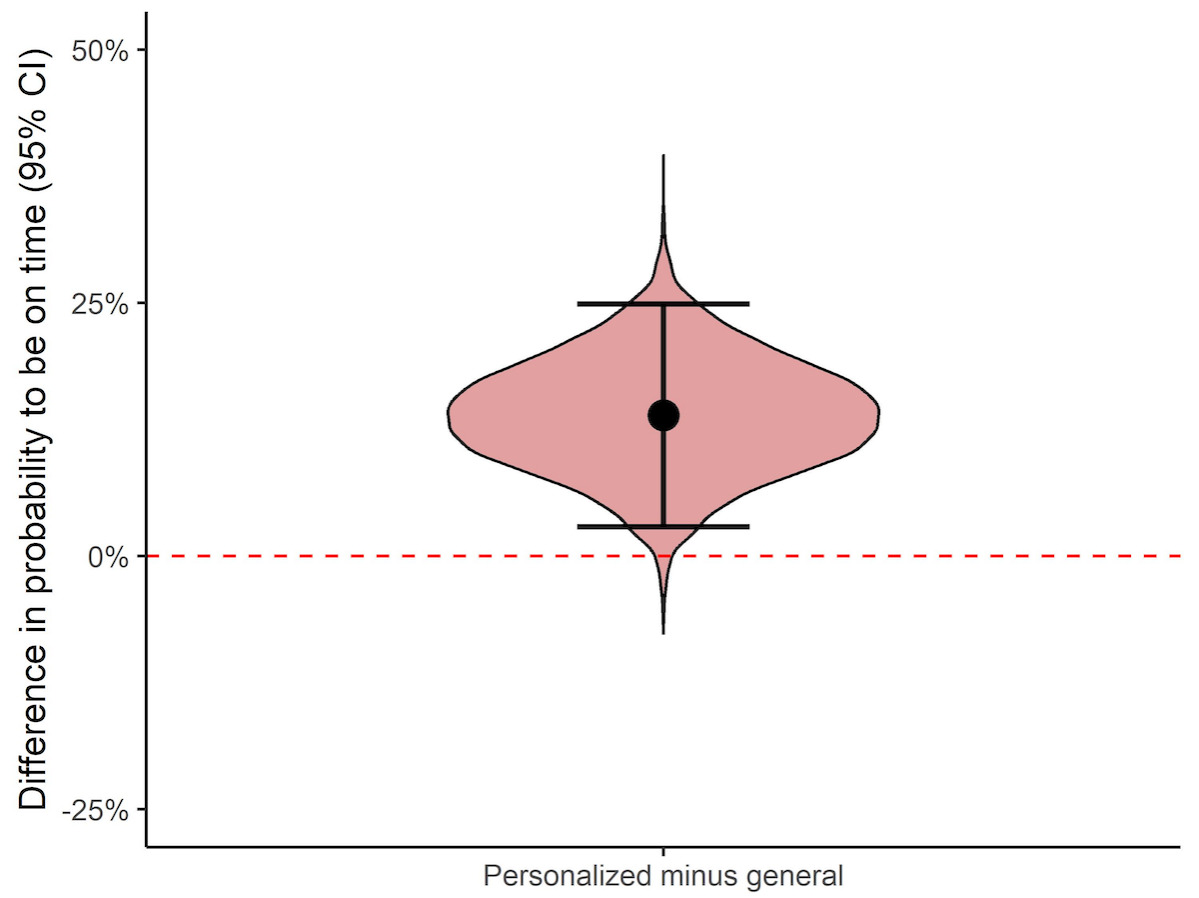
Violin plot, median difference, and 95% credible interval (CI) illustrating the estimated difference in the probability to be on time with the course (personalized reminders minus general reminders). The width of the violin plot illustrates the relative probability of possible differences in probabilities for being on time.

By simply inspecting the data and comparing the number of times participants were on time following the two types of reminders, it was clear that the probability of being on time with one’s studies was higher when the participants received personalized reminders than when they received general reminders (Table 1). Following personalized reminders, the probability of being on time was 64%, whereas the probability was 53% following general reminders. Thus, the absolute difference in the probability to be on time was 11 percentage points higher following a personalized reminder. However, although the difference is clear by simply comparing the two reminder types, this analysis does not consider the effects of individuals and reminder times on the probability to be on time. There are likely interindividual differences in the probability to be on time, and because the challenges to being on time could vary during the 6 months over which the course ran, it would be advantageous to include this information in the analysis. Therefore, as mentioned in the Data Analysis section, a Bayesian mixed-effects logistic regression was used, which considered both interindividual differences and differences between reminder times when estimating the impact that the reminders had on participants’ probability of being on time. As can be seen in Figure 2, the analysis indicated that the probability of being on time for an average participant at an average reminder time was higher following personalized reminders (70%, 95% CI 51%-85%) than following general reminders (56%, 95% CI 37%-73%). Specifically, the estimated difference in probability was 14 percentage points higher (95% CI 3%-25%) for personalized reminders compared to general reminders (Figure 3). However, there is some uncertainty about the size of the benefit of personalized over general reminders, as the CI indicates that there is a benefit of personalized reminders, but the effect may be as small as 3 percentage points or as large as 25 percentage points. Nevertheless, the median estimated difference indicates that, if 100 students received personalized reminders, 14 more students would be on time with their coursework than if they received general reminders.

**Table 1:** The number of times a reminder was followed by a participant being on time when reminders were general or personalized.

Reminder type	On time, n (%)	
	No	Yes
General (n=390)	184 (47.2)	206 (52.8)
Personalized (n=195)	70 (35.9)	125 (64.1)

## Discussion

### Principal Findings

We compared the effect of personalized email reminders to simple general reminders in an online program with 39 adult professionals. The personalized reminders had a positive effect on the timeliness of the participants as compared to the general reminders. Based on our results, if 100 students were given personalized reminders instead of general reminders, we would expect that an additional 14 students would be on time with their coursework. Considering how small the intervention was—adding a few words and information on which page the student ought to be on to an email reminder—we consider this effect fairly substantial.

Being on time in online courses is beneficial for both students and teachers. However, in adult education settings where the students are working full time, it is also important to give the students freedom to study when they can to allow them to handle variations in workload and family life. One approach is to allow for a long calendar time to work with the learning material individually and then meet at a synchronized time for discussions and group work. When performing group work, it is important that all students have at least a comparable knowledge base of the work at hand, which means that, on those occasions during a course, all students should be in sync or at least have reached a minimum level of understanding of the subject to be able to contribute to the group work.

### Comparison With Prior Work

Reminders can be a tool to improve the timeliness of students’ study behavior [2,3,7,16,17], but have different effects on different students and may, in some cases, even reduce timeliness [9,18]. Therefore, it is important to examine which type of reminders work for the intended target group before using reminders at a large scale.

Reminders can be viewed as a feedback intervention on the learners’ progress in the course. As such, it has been suggested that reminders should focus on previously established goals, rather than the self [19]. However, researchers have argued that people compare themselves to others to establish their personal worth, in line with social comparison theory [6], and therefore focused reminders on the self [8]. In a MOOC study, reminders were added to the individuals’ course pages. The reminders compared the students’ performance with that of other previously successful students. They varied the feedback by combining either individualistic (referring to the students as individuals) or collectivistic (referring to the course collective) expressions with promotional or preventative feedback. Both types of feedback improved the final grade, but only for learners who had a bachelor’s degree or higher [7].

Email reminders made students in an online medical terminology course score better on their weekly quizzes [20]. On the contrary, no significant impact on grades or attitude was found in a study focusing on online nursing university courses [4]. These differences in results may be due to differences in study design and potential bias, but it is also important to look at the study participants and their backgrounds. Most studies focus on college and university students, whereas our study was directed toward adult learners working full-time.

An email intervention in an international MOOC to counter procrastination in taking the final test had mixed results depending on the participants’ stated country. A previous version of the MOOC showed a correlation between taking the test early and higher grades, and when sending reminders, the effect varied by country, with participants from Germany performing 167% better (in qualifying for a certificate) than the control group. Conversely, reminders had no statistically significant effect on the ability of participants from Nigeria to qualify for a certificate; rather, it increased their procrastination [18].

How successful reminders are may depend on how they are designed. A study of email versus SMS text message reminders found that SMS text message reminders improved the time in which students turned in assignments, their confidence, their mastery of the course material, and the overall class performance [21]. Students often ignored emails [22], and students who signed up to receive SMS text messages had better course performance and submitted more assignments on time as compared to students who just received email reminders [20]. In addition, SMS text message reminders may have a stronger (but temporary) effect on students with low socioeconomic status [23]. However, the results on SMS text message reminders are mixed in the literature. Many studies have allowed the participants to choose this option, which creates a bias. In this study, the technical challenges with distributing SMS text messages from Sweden to three East African countries were considered too high, and therefore, we decided to use email reminders.

Most East African countries suffer from inadequately skilled labor, and its availability is unevenly distributed geographically, with most skilled people in cities [24]. Governments have adopted digital technologies as a key strategy to achieve their educational priorities [25]. Online learning can be an effective and efficient approach for disseminating specialized education programs in low- and middle-income countries [26], as employed adult learners with families and other out-of-work responsibilities can enroll in online education programs with a flexible schedule [27]. This increased flexibility is an important possibility in low- and middle-income countries [28].

### Limitations

Our study was limited in numbers with only 39 participants. All participants came from three neighboring East African countries and had at least a bachelor’s degree. However, their educational background was diverse and spanned public health, nutrition, environmental health, medicine, agriculture, sociology, and chemistry. As several previous studies have shown [4,7,9,18,20,23], it is important to study the effect of reminders in each cultural context before implementing them on a large scale. We only studied the effect over 3 2-month periods for part-time adult learners and can only speculate on how this intervention would work in shorter or longer courses, for different groups of students, or at different study paces.

### Strengths

Despite the limited number of participants, we saw a substantial effect on timeliness. The intervention we used was independent of any particular system, so it can be repeated manually by anyone, at least in courses of similar student size. For larger courses, anyone with basic programming skills can create a semiautomated data flow that takes the progress data, the schedule, and the students’ email addresses as input and generates and distributes the email reminders.

### Conclusions

After experiencing challenges with procrastination in the first OneLearns installment in 2021, which affected the group work assignments negatively, we decided to examine whether personal reminders would help compared to the previous general reminders. Our conclusion is that the personal reminders improved the situation substantially but did not eliminate procrastination completely.

## Appendix

Multimedia Appendix 1 Python program to convert percentage of performance into page numbers.

Multimedia Appendix 2 CONSORT-EHEALTH checklist (V 1.6.1).

## Abbreviations

CI: credible interval
MOOC: massive open online course
NGO: nongovernmental organization

## Data Availability

The data can be requested from KTH’s Registrar's Office [29].
