# Supplementary material for: Effect of Personalized Email-Based Reminders on Participants’ Timeliness in an Online Education Program: Randomized Controlled Trial
Source: JMIR Form Res. 2023 Oct 13;7:e43977. doi: 10.2196/43977 (PMC10611998; doi:10.2196/43977)
Supplement: Multimedia Appendix 1 [file formative_v7i1e43977_app1.pdf]

```python
""" Reads a file with percentages of activities and maps that to pages in
OneLearns.
"""
PAGE_TO_BE_ON=49
#FIRST_PAGE_IN_MODULE=1 #M1
#FIRST_PAGE_IN_MODULE=6 #M2
#FIRST_PAGE_IN_MODULE=19 #M3
#FIRST_PAGE_IN_MODULE=28 #M4
FIRST_PAGE_IN_MODULE=42

#CUMULATIVE_PERCENTAGES = [56, 100]  # M1
#CUMULATIVE_PERCENTAGES = [4, 7, 11, 24, 38, 53, 73, 82, 89] # M2
#CUMULATIVE_PERCENTAGES = [12, 24, 39, 59, 66, 76, 88, 100] # M3
#CUMULATIVE_PERCENTAGES = [2, 15, 24, 32, 44, 51, 63, 71, 80, 90, 100] # M4
CUMULATIVE_PERCENTAGES = [6, 13, 27, 42, 49, 53, 59, 88, 100] # M5

DEFAULT_FILENAME = "OneLearnsF21.csv"

def find(n):
    for i in range(len(CUMULATIVE_PERCENTAGES)):
        if (n<CUMULATIVE_PERCENTAGES[i]):
            return i
    return len(CUMULATIVE_PERCENTAGES)

def läs_filnamn(prompt):
    """Läser ett filnamn till dess att filen går att öppna"""
    fil_öppnad = False
    while not fil_öppnad:
        s = input(prompt)
        if s=="":
            s = DEFAULT_FILENAME
        filnamn = s
        try:
            fil = open(filnamn, 'r')
            fil_öppnad = True
        except FileNotFoundError:
            print(filnamn, "existerar inte (i den katalogen). Vänligen försök
igen.")
    return fil


fil = läs_filnamn("Filename of activities: (default: "+DEFAULT_FILENAME+") ")

studentlist = fil.readlines()

for s in studentlist[5:]:
    last_name, first_name, position, irrelevant = s.split(",")
    if position == "0%":
        print(last_name, first_name, "Nothing here, see previous module.")
    elif position == "100%":
        print(last_name, first_name, "Done with this module, see next module.")
    else:
        p = int(position[:-1])
        i = find(p)+FIRST_PAGE_IN_MODULE
```

```
    if i<PAGE_TO_BE_ON:
        print(last_name, first_name, "is behind on page", i)
    else:
        print(last_name, first_name, "is on time on page", i)
#print("Månad%2d: %8.2f kr %6.2f kr" % (m, sparkapital, ökning))
```
